# Exome Sequencing Identifies Early Gastric Carcinoma as an Early Stage of Advanced Gastric Cancer

**DOI:** 10.1371/journal.pone.0082770

**Published:** 2013-12-23

**Authors:** Guhyun Kang, Woo Cheol Hwang, In-Gu Do, Kai Wang, So Young Kang, Jeeyun Lee, Se Hoon Park, Joon Oh Park, Won Ki Kang, Jiryeon Jang, Min-Gew Choi, Jun Ho Lee, Tae Sung Sohn, Jae Moon Bae, Sung Kim, Min Ji Kim, Seonwoo Kim, Cheol Keun Park, Kyoung-Mee Kim

**Affiliations:** 1 Department of Pathology, Samsung Medical Center, Sungkyunkwan University School of Medicine, Seoul, Korea; 2 Oncology Research Unit, Pfizer Inc., San Diego, California, United States of America; 3 Department of Medicine, Division of Hematology-Oncology, Samsung Medical Center, Sungkyunkwan University School of Medicine, Seoul, Korea; 4 Department of Surgery, Samsung Medical Center, Sungkyunkwan University School of Medicine, Seoul, Korea; 5 Samsung Advanced Institute for Health Sciences & Technology, Sungkyunkwan University School of Medicine, Seoul, Korea; 6 Biostatistics Unit, Samsung Biomedical Research Institute, Samsung Medical Center, Seoul, Korea; 7 Department of Pathology, Sanggye Paik Hospital, Inje University College of Medicine, Seoul, Korea; Duke-National University of Singapore Graduate Medical School, Singapore

## Abstract

Gastric carcinoma is one of the major causes of cancer-related mortality worldwide. Early detection and treatment leads to an excellent prognosis in patients with early gastric cancer (EGC), whereas the prognosis of patients with advanced gastric cancer (AGC) remains poor. It is unclear whether EGCs and AGCs are distinct entities or whether EGCs are the beginning stages of AGCs. We performed whole exome sequencing of four samples from patients with EGC and compared the results with those from AGCs. In both EGCs and AGCs, a total of 268 genes were commonly mutated and independent mutations were additionally found in EGCs (516 genes) and AGCs (3104 genes). A higher frequency of C>G transitions was observed in intestinal-type compared to diffuse-type carcinomas (*P* = 0.010). The *DYRK3, GPR116, MCM10, PCDH17, PCDHB1, RDH5* and *UNC5C* genes are recurrently mutated in EGCs and may be involved in early carcinogenesis.

## Introduction

Gastric carcinoma (GC) is a heterogeneous disease with multiple environmental etiologies, alternative pathways of carcinogenesis and no known high-frequency oncogenic perturbation [1], [2], [3]. The Lauren classification has proven useful in evaluating the natural history of GC, especially with regard to incidence trends, clinicopathologic correlations and etiologic precursors [4]. Lauren classified gastric adenocarcinoma into intestinal and diffuse according to morphological features of the tumor [4], [5], [6]. Intestinal-type carcinomas are believed to arise secondary to chronic atrophic gastritis associated with *H. pylori* and intestinal metaplasia [7]. Diffuse-type GCs are not associated with intestinal metaplasia and may arise from single-cell mutations within normal gastric glands [4], [8], [9].

GC is one of the major causes of cancer-related mortality worldwide. Early detection and treatment results in an excellent prognosis for patients with early gastric cancer (EGC), whereas the prognosis of patients with advanced gastric cancer (AGC) remains poor. However, it is unclear whether EGCs and AGCs are distinct entities or are the same tumor progressing from early to advanced stages [10]. The molecular signatures distinguishing EGC from AGC are important to aid identification of novel prognostic markers and potential therapeutic targets.

Recently, exome sequencing in 22 [11] and 15 [12] AGC samples showed frequent inactivating mutations in cell adhesion and chromatin-remodeling genes, and the genetic alterations differed among subgroups stratified by Epstein-Barr virus (EBV) or *H. pylori* infection and microsatellite instability (MSI) status. To further explore the genetic alterations underlying GCs, we performed whole exome sequencing in four matched pairs of EGC and normal tissue, and compared the results to those from AGCs.

## Materials and Methods

### Sample preparation

Tumor and non-neoplastic gastric tissues were collected from gastrectomy specimens. The present study was conducted after the approval from the Institutional Review Board of Samsung Medical Center, and all patients gave written informed consent prior to surgery. For tumor samples, masses were >4 cm on gross inspection, and the surface mucosa from each tumor was procured. After embedding in OCT media, the tissue was cut and H&E stained. Samples of >90% tumor content were selected for DNA extraction with a Mini Kit (Qiagen, Valencia, CA, USA) and treated with RNase A to remove remaining RNA. DNA was also extracted from paired unaffected gastric tissue, which was obtained distant from the tumor site and confirmed to be tumor-free. MSI was analyzed with five NCI markers as previously described [13]. The presence of EBV was detected by EBV-encoded RNA *in situ* hybridization as previously described, and only cases with strong signal within almost all of the tumor cell nuclei were considered positive [14]. Additional details for the EGC samples are provided in Table 1.

**Table 1:** Clinicopathologic data of early gastric cancers.

Case No.	Gender/Age(yr)	EBV status	Microsatellite instability	Tumor site	Histologic type	Lauren's classification	TNM stage
1	M/70	negative	stable	antrum	tubular adenocarcinoma, well differentiated	intestinal	T1bN0M0
2	M/53	positive	stable	body	tubular adenocarcinoma, moderately differentiated	intestinal	T1bN1M0
3	F/73	negative	high	antrum	signet ring cell carcinoma	diffuse	T1bN0M0
4	F/50	negative	stable	body	signet ring cell carcinoma	diffuse	T1bN0M0

T1b, Tumor invasion to the submucosa; N0, No regional lymph node metastasis; N1, Metastasis in 1 to 2 regional lymph nodes; M0, No distance metastasis.

### Exome enrichment and sequencing

Exome enrichment (SureSelect Human All Exon Kit, Agilent Technologies) and Illumina sequencing libraries were prepared according to the manufacturer's instructions. Briefly, 3 μg of genomic DNA was sheared with the Covaris S2 system; the DNA fragments were end-repaired, extended with an ‘A’ base on the 3′ end, ligated with paired-end adaptors and amplified (four cycles). Exome-containing adaptor-ligated libraries were hybridized for 24 h with biotinylated oligo-RNA baits and enriched with streptavidin-conjugated magnetic beads. The final libraries were further amplified through 11 PCR cycles and subjected to Illumina sequencing on one lane of the HiSeq 2000 sequencer with a targeted insert size of ∼180 bp. All sequencing was run with paired-end 65-bp reads and was performed according to Ilumina's standard protocol. On average, ∼136.3 million purity-filtered reads were generated for each sample. The mean percentage of duplicate reads due to PCR and optical artifacts was 0% in our data set, and ∼123.7 million uniquely mapped reads were obtained for each sample. On average, 69.1% of reads in each sample had at least 50% overlap with any targeted region ±100 bp in the SureSelect whole exome bait library. The targeted regions in each sample were sequenced to an average depth of 113.7×, with ∼98.8% of the targeted regions covered ≥1×, ∼94.3% ≥10×, ∼82.4% ≥30×, ∼70.8% ≥50×, ∼66.4% ≥60×, ∼62.2% ≥70×, ∼58.2% ≥80×, ∼54.4% ≥90× and ∼50.8% ≥100×. Detailed summaries of raw data quality are described in Table S1. For comparison, the same algorithm (SMART), used in the previous dataset of AGC samples [11], was applied to these data to identify somatic single-nucleotide variations and insertions/deletions (indels) alterations from short read sequencing data. The data set has been deposited in the European Nucleotide Archive and can be accessed at http://www.ebi.ac.uk/ena/data/view/PRJEB 4850.

Mutations detected by exome sequencing were further validated by PCR and Sanger sequencing. Briefly, primers are designed using Primer3 software (http://frodo.wi.mit.edu), and the sequences are listed in Table S3. The PCR-amplified products were then sequenced using a BigDye Terminator v3.1 Cycle Sequencing Kit and an ABI 3700 automated sequencer (Applied Biosystems, Foster City, CA, USA).

## Results

### Somatic alterations in EGCs

**Figure 1:**
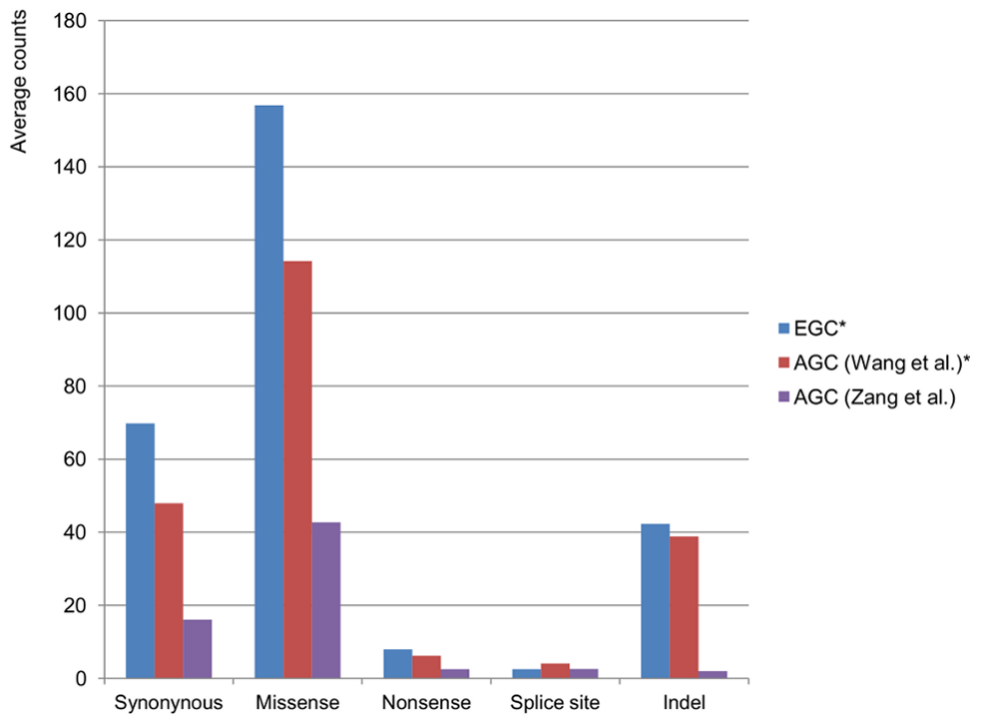
Mutation spectra of early and advanced gastric cancers. The star (*) indicates results produced on the same platform.

**Figure 2:**
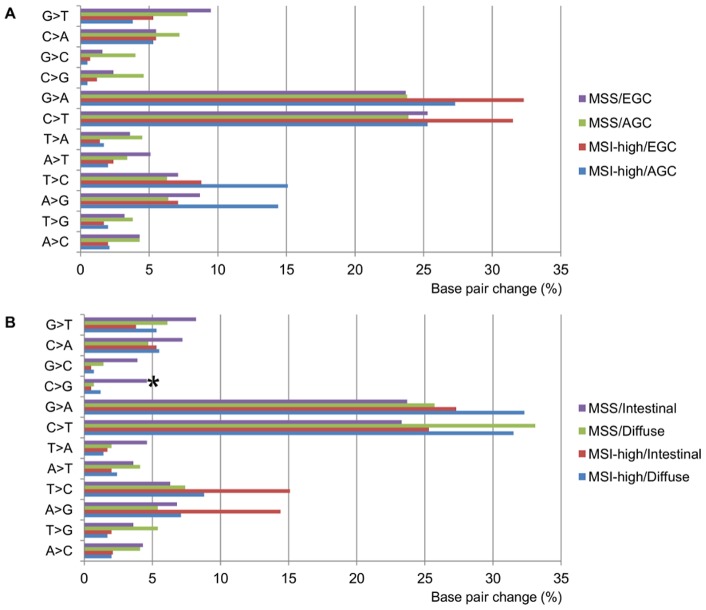
Mutation spectra of gastric cancers according to pathologic stage, microsatellite instability status and histologic classification. The star (*) indicates significant difference between intestinal- and diffuse-type in all microsatellite stable cancers (*P* = 0.010).

In total, 2,389 somatic mutations were identified in the four EGC samples, of which 1,117 occurred in coding regions or essential splice sites (627 missense, 32 nonsense, 10 essential splice site, 169 indels and 279 synonymous) (Figure 1, and Tables 2 and S2). One GC with MSI-high had 727 non-silent mutations including mismatch repair genes (*MSH6* and *MSH3*), whereas the three microsatellite stable (MSS) samples had an average of 37, a difference of approximately 20-fold. The nonsynonymous-to-synonymous ratios in the MSS cancers tended to be higher than that of the MSI-high cancer, but the difference was not statistically significant. C>T and G>A transitions were the most common mutation (61%) in the EGCs, and there was no significant difference in single base pair changes between MSI-high and MSS cancers (Figure 2A and Table S4). Of 784 genes harboring non-silent mutations, 13 were mutated in two or more samples. These included genes known to be involved in gastric carcinogenesis (*TP53*) and reported in the Catalogue of Somatic Mutations in Cancer (COSMIC) to be mutated in GCs (*DYRK3, MCM10, PCDH17* and *UNC5C*) (Table 3). Of the genes selected for validation, *PCDH17* mutation was most likely not validated by Sanger method because of low frequencies of mutant allele (Table S3). Interestingly, in a diffuse-type EGC with MSI-high, an *EGFR* (c.2224G>A, p.V742I) mutation was identified.

**Table 2:** Summary of somatic mutation types and prevalence in early gastric cancers.

Case No.	Missense	Stop gained	Stop lost	Essential splice site	Synonymous	Insertion/Deletion	Total	Nonsynonymous/Synonymous ratio
1	58	3	0	1	20	1	83	3.05
2	28	1	0	0	10	0	39	2.90
3	526	26	0	9	244	166	971	2.26
4	15	2	0	0	5	2	24	3.40
Overall total	627	32	0	10	279	169	1117	2.36

**Table 3:** List of genes with protein-altering mutations in at least two early gastric cancer samples.

Gene symbol	Selected biological process/ molecular function terms*	No. of mutated samples	SNVs/indels in MSS	SNVs/indels in MSI-high	No. of background mutations
*BCORL1*	DNA-dependent regulation of transcription, chromatin modification	2	c.4397G>A	c.5036delC	0
*DYRK3*	erythrocyte differentiation, protein phosphorylation, protein kinase activity	2	c.557A>G	c.130delC	0
*GPR116*	G-protein coupled receptor activity, neuropeptide signaling pathway	2	c.2731G>C, c.2276G>A		0
*LRP2*	cell proliferation, endocytosis, protein glycosylation, lipid metabolic process	2	c.13210C>T	c.4345C>T	2
*LRP12*	regulation of growth, signal transduction, endocytosis	3	c.2110G>C, c.1523A>G	c.1351delA	0
*MACF1*	Wnt receptor signaling pathway, cell cycle arrest, cellular component movement	2	c.5789G>T	c.200G>A	0
*MCM10*	DNA replication, cell cycle checkpoint	2	c.650C>T	c.1789C>T	0
*PCDH17*	homophilic cell adhesion	2	c.1549G>A, c.1738G>A		0
*PCDHB1*	homophilic cell adhesion	2	c.1547C>T	c.5C>T	0
*PRKCI*	cell-cell junction organization, cytoskeleton organization, regulation of NF-kappaB transcription factor activity	2	c.772C>T	c.819delA	0
*RDH5*	response to stimulus, retinol metabolic process	2	c.73C>G	c.712delG	0
*TP53*	cell cycle checkpoint, DNA damage response, regulation of apoptotic process, cell differentiation	2	c.736T>C	c.743C>T	0
*UNC5C*	apoptotic process, regulation of cell migration	2	c.1006G>A	c.1508delG	0

* Provided by UniProt-GOA.

SNV, single nucleotide variation; indels, small insertion or deletion; MSS, microsatellite stable; MSI-high, high level of microsatellite instability.

### Comparison between EGC and AGC

For comparison of our results on EGC with those of AGCs, two recently published whole exome sequencing data were used [11], [12]. Wang *et al*. detected 164 non-silent and 48 synonymous mutations on average in 22 AGC samples with 116× average coverage depth [11]. Zang *et al*. detected on average 50 non-silent and 16 synonymous somatic mutations in 15 AGC samples with 96× average coverage depth [12]. In direct comparison between the four EGCs and 37 AGCs, there was no significant difference in the numbers of mutation type (Figure 1). The single base pair changes in EGCs were similar to a previous report by Wang *et al*. [11], showing a distinctly higher number of C>T and G>A transitions in both MSS and MSI-high tumors (Figure 2A and Table S4). Interestingly, C>G transitions were more common in intestinal-type than in diffuse-type GCs across all MSS samples, which included three EGCs and 18 AGCs (Wilcoxon rank sum test, *P* = 0.010) (Figure 2B and Table S4).

**Figure 3:**
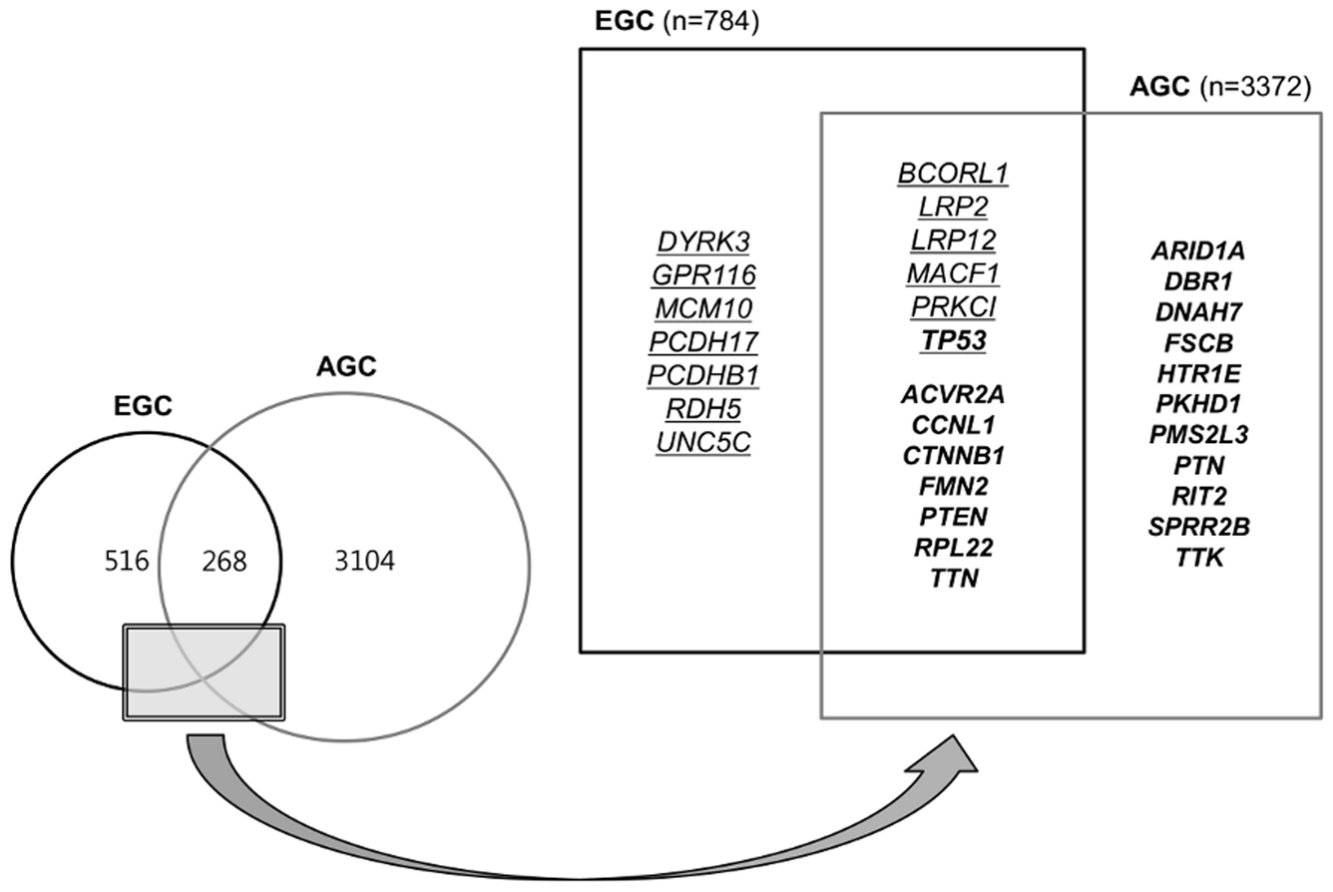
Venn diagram and schematic representation of all genes with non-silent mutations in gastric cancers. Underlined and bold font indicates the genes with protein-altering mutations in at least two early cancer samples and the selected genes with higher-than-expected mutation rates in advanced gastric cancers (false discovery rate<0.2), respectively.

In 37 AGC and 4 EGC samples, non-silent mutations (missense, nonsense, essential splice site and indels) were detected in 3,372 and 784 genes, respectively. In both EGCs and AGCs, 268 genes were commonly mutated; the *BCORL1, LRP2, LRP12, MACF1, PRKCI* and *TP53* genes were mutated in at least two EGC samples, and the *ACVR2A, CCNL1, CTNNB1, FMN2, PTEN, RPL22* and *TTN* genes, as well as others, were significantly associated with AGCs with a false discovery rate of <0.2 [11],[12] (Figure 3). Functional annotation analysis using DAVID (http://david.abcc.ncifcrf.gov) to examine the genes found overlap between the two sample sets revealed that the significantly enriched terms included actin binding, cytoskeleton, cell projection and cell-cell junction (Table S5).

## Discussion

Although whole exome sequencing has been reported for 37 AGC samples [11], [12], there has been no such study to evaluate early carcinogenesis at the genetic level. To explore the complete repertoire of somatic mutations in EGCs, we performed whole exome sequencing of four paired EGC samples, and found distinct and common genetic signatures between EGCs and AGCs that may identify genes involved in early carcinogenesis and subsequent progression.

Epithelial cancers often have variable mutation spectra pointing to particular mutagenic stimuli [15], [16]. For example, high rates of A>C and C>T transitions were observed in esophageal adenocarcinomas and sun-exposed melanomas, respectively, suggesting that these mutations are attributable to gastroesophageal reflux and ultraviolet exposure [15], [17]. A previous genome-wide sequencing study in two gastric adenocarcinomas showed frequent C>A and T>A alterations compared to normal genomes [18]. Here, we found frequent C>G transitions in intestinal-type carcinomas compared to diffuse-type GCs after exclusion of MSI-high GCs. Our unique observation warrants future studies to define specific etiology that potentially contributes to understanding of the complex and poorly understood molecular pathways of intestinal-type GCs.

Through comparative analysis, we identified 268 overlapping genes with non-silent mutations shared by both EGCs and AGCs (Figure 3). About one-third of the non-silent mutations in EGCs are shared with AGCs and 8% of the non-silent mutations found in AGCs are shared with EGCs. A previous study with gene expression analysis showed that the majority of alterations associated with EGCs are retained in AGCs and further expression changes mark the transition from EGC to AGC [10]. Overall, these results indicate that EGC represents an early molecular stage of AGC, and the commonly mutated genes play important roles in the progression from EGC to AGC. We reconfirmed that *TP53* is the most frequently mutated gene in GCs, with *TP53* mutations found in half of EGC and two-thirds of AGC samples. Among the overlapping genes, *AKAP9, CAMTA1, COL1A1, CTNNB1, KDM5A* and *RPL22* were annotated as oncogenes, whereas *ATM, FBXW7, MSH6, NF1, PTEN, SETD2* and *TP53* were tumor suppressor genes by the Sanger Gene Census (http://cancer.sanger.ac.uk/cancergenome/projects/census). Of the Cancer Census genes, we first identified an *EGFR* mutation (c.2224G>A, p.V742I) in a diffuse-type EGC with MSI-high. In a recent study on 63 MSI-high GCs, *EGFR* mutation was not detected by direct sequencing of the kinase domain (exons 18, 19, 20 and 21) [19]. The same V742I mutation has been reported in a patient with endometrial cancer and in a glioma cell line [20], [21]. The clinical significance of this rare mutation needs to be validated in the near future.

Although the prevalence of recurrent mutations in EGCs was relatively low, 13 genes were mutated in at least two samples, and had very few synonymous, intronic and/or untranslated mutations. Among these 13 genes, *DYRK3, GPR116, MCM10, PCDH17, PCDHB1, RDH5* and *UNC5C* may be specific for early stage GC, suggesting a possible role in the early carcinogenesis. In our series, *PCDH17* mutations occurred in intestinal-type GCs with MSS, including one EBV-positive sample. Previous global genomic analyses of colorectal and pancreatic cancers also revealed missense mutations in some members of *PCDH* (protocadherin) subfamilies [22], [23]. However, the mutations detected in our EGCs by Illumina sequencing were not confirmed by Sanger sequencing, probably because the mutant allele frequencies were very low. *UNC5C* belongs to the functional dependence receptor family, members of which share the ability to induce apoptosis in the absence of their ligands [24], [25]. Aberrant methylation of this gene has been reported in the course of gastric carcinogenesis, and this methylation disappeared in highly advanced GCs [26]. For the remaining genes, their functional relevance in GC remains unclear.

Loss of function in cell adhesion molecules increases the ability of tumor cells to invade surrounding tissue, and dysfunction in chromatin-remodeling complex promotes chromosomal instability that drives tumorigenesis [27]. None of our EGC samples had protein-altering mutations of chromatin-remodeling genes found in AGCs, such as *ARID1A, MLL3, PBRM1* and *MBD2* [11], [12], suggesting chromatin modification occurs late in the progression of GC.

Overall, our study suggests that EGC and AGC share common somatic mutations, and AGC is associated with additional cumulative genetic alterations in cell adhesion and chromatin-remodeling genes. The molecular signatures distinguishing EGC from AGC are important to help identify novel prognostic markers and potential therapeutic targets. Larger studies are needed to determine the biologic significance of the recurrently mutated genes in EGCs.

## Supporting Information

Table S1 Summary of whole exome sequencing statistics for four pairs of gastric samples.
(PDF)

Table S2 List of all somatic mutations in early gastric cancers identified by exome sequencing.
(XLS)

Table S3 Primers for Sanger sequencing, allele frequency and validation results.
(PDF)

Table S4 Comparison of somatic point mutation spectra in gastric cancers by tumor state and histologic type.
(PDF)

Table S5 DAVID pathway analysis of the 268 overlapping genes between early and advanced gastric cancers.
(XLS)
