# Supplementary material for: Exome Sequencing Identifies Early Gastric Carcinoma as an Early Stage of Advanced Gastric Cancer
Source: PLoS One. 2013 Dec 23;8(12):e82770. doi: 10.1371/journal.pone.0082770 (PMC3871845; doi:10.1371/journal.pone.0082770)
Supplement: Table S1 — Summary of whole exome sequencing statistics for four pairs of gastric samples. (PDF) [file pone.0082770.s001.pdf]

Supplementary Table 1. Summary of whole exome sequencing statistics for four pairs of gastric samples.

Case No.	Read length	Total PF reads	Total HQ uniquely mapped reads	# reads in targeted regions +/-100bp (min fraction: 0.5)	% reads in targeted regions +/-100bp (min fraction: 0.5)	# bases in targeted regions	% bases in targeted regions	Mean coverage	Median coverage	>=1X	>=10X	>=30X	>=50X	>=100X
Normal 1	65	129,997,828	119,763,389	85,261,792	71.2%	4,315,720,151	53.0%	114.7	85	98.4%	92.2%	78.2%	66.4%	44.0%
Tumor 1	65	122,585,721	111,165,743	75,938,176	68.3%	3,833,073,700	50.7%	101.9	84	98.8%	94.3%	82.4%	69.9%	41.7%
Normal 2	65	161,928,120	147,184,937	100,785,607	68.5%	5,083,417,090	50.8%	135.1	102	98.3%	91.9%	79.5%	69.7%	50.6%
Tumor 2	65	156,989,079	143,373,596	98,147,306	68.5%	4,985,278,129	51.1%	132.5	103	98.5%	92.9%	80.9%	70.8%	50.8%
Normal 3	65	134,002,492	122,308,791	82,945,029	67.8%	4,218,519,377	50.7%	112.1	92	98.6%	93.7%	81.7%	70.4%	46.3%
Tumor 3	65	95,756,622	87,306,451	59,928,090	68.6%	3,056,917,957	51.5%	81.2	68	98.6%	92.8%	77.2%	62.2%	31.2%
Normal 4	65	145,845,854	129,594,610	91,136,558	70.3%	4,370,516,184	51.9%	116.2	90	98.2%	91.5%	78.0%	67.2%	46.2%
Tumor 4	65	143,731,855	128,968,300	90,200,830	69.9%	4,356,431,540	52.0%	115.8	92	98.3%	92.1%	79.0%	68.2%	46.5%
Average		136,354,696	123,708,227	85,542,924	69.1%	4,277,484,266	51.5%	113.7	89.5	98.5%	92.7%	79.6%	68.1%	44.7%
