# Supplementary material for: Exome Sequencing Identifies Early Gastric Carcinoma as an Early Stage of Advanced Gastric Cancer
Source: PLoS One. 2013 Dec 23;8(12):e82770. doi: 10.1371/journal.pone.0082770 (PMC3871845; doi:10.1371/journal.pone.0082770)
Supplement: Table S3 — Primers for Sanger sequencing, allele frequency and validation results. (PDF) [file pone.0082770.s003.pdf]

Supplementary Table 3. Primers for Sanger sequencing, allele frequency and validation results.

Gene	Chromosome	Position	Reference base	Mutant base	Forward	Reverse	Allele frequency (%)*	Sanger confirmation
BCORL1	X	129171433	G	A	GTCCCCACAGGATGTTGTTC	GGGAGATGCAGCCCTCTT	47	Yes
DYRK3	1	206821100	A	G	GCAGATGGGGCCTATATTCA	CAGCTGCTTGACGATGAAAG	20	Yes
GPR116	6	46828555	G	A	AGAGCCCCTCTCAGGATGAG	CAGAGAAGCTGCAATGGGGA	10	No
GPR116	6	46826909	G	C	CTCTGGGGCAATGTGGTCAT	TTGGCAAGCCTGAAGTTCCA	29	Yes
LRP2	2	170094762	C	T	TGAACAGCATCTGAGAGTCTGC	TTTTGAAACGCACTCCAGGT	19	Yes
LRP2	2	169996110	C	T	GTTTGCATGGTGTGGGCAC	TTTCCCCAGAAGAGGCAAGG	17	Yes
LRP12	8	105503371	G	C	TTGAGGCCCAATAGTTCTGTC	GTGATGGCAGCGATGAAGA	27	Yes
LRP12	8	105509257	A	G	TTGAGGCCCAATAGTTCTGTC	GTGATGGCAGCGATGAAGA	6	No
MACF1	1	39806513	G	T	AGTGTTAAATCAGCACACACAGC	ACTGTTAGATCCCTTGCTGTTC	22	Yes
MACF1	1	39550090	G	A	AGTGTTAAATCAGCACACACAGC	ACTGTTAGATCCCTTGCTGTTC	16	Yes
MCM10	10	13217564	C	T	TCAGTGTGGTTGCCTTTTCA	GGGGTCCCCACAATTTGAC	14	Yes
MCM10	10	13237081	C	T	TTTCTGCAGAGCTCAAGTGAAG	CTCCAGCCTGGGGAACTC	20	Yes
PCDH17	13	58208229	G	A	AGTACCTGGGCTCTGTGCTC	CTCAAAAGCCTTGGTCTGCT	6	No
PCDH17	13	58208418	G	A	AGTACCTGGGCTCTGTGCTC	CTCAAAAGCCTTGGTCTGCT	13	No
PCDHB1	5	140432602	C	T	GACCACCTAGCTTGTCTGCC	CGTAGAGCTTCCCATTGCCT	24	Yes
PCDHB1	5	140431060	C	T	ACCTGTTGCAGAAAAGTGAAAGT	AAACGAGCCGCTCTCCATTT	15	Yes
PRKCI	3	169998081	C	T	CAACAGGCAATGAACACCAG	TTTTTCACAACTTTCATTGCATAA	9	Yes
RDH5	12	56115041	C	G	GTAGGTCACTTGGGCTCCAG	GAGGGGGTCAGGCAGCTG	17	Yes
TP53	17	7577538	C	T	TGGCTCTGACTGTACCACCA	GGGTCAGAGGCAAGCAGA	16	Yes
TP53	17	7577545	T	C	TGGCTCTGACTGTACCACCA	GGGTCAGAGGCAAGCAGA	20	Yes
UNC5C	4	96163682	G	A	CATCCCTTAAGCAGCTGCCT	CTGCATGCAAAGCCCATCAG	22	Yes

*Allele frequency is the proportion of variant reads present in the exome sequencing data for the tumor.
