# Supplementary material for: Exome Sequencing Identifies Early Gastric Carcinoma as an Early Stage of Advanced Gastric Cancer
Source: PLoS One. 2013 Dec 23;8(12):e82770. doi: 10.1371/journal.pone.0082770 (PMC3871845; doi:10.1371/journal.pone.0082770)
Supplement: Table S4 — Comparison of somatic point mutation spectra in gastric cancers by tumor state and histologic type. (PDF) [file pone.0082770.s004.pdf]

Supplementary Table 4. Comparison of somatic point mutation spectra in gastric cancers by tumor stage and histologic type.

Base pair change	Early cancer	Advanced cancer	Intestinal / Mixed	Diffuse-type
MSS				
A>C	11 (4.3%)	75 (4.3%)	80 (4.3%)	6 (4.1%)
T>G	8 (3.2%)	67 (3.8%)	67 (3.6%)	8 (5.4%)
A>G	22 (8.7%)	111 (6.4%)	125 (6.8%)	8 (5.4%)
T>C	18 (7.1%)	109 (6.3%)	116 (6.3%)	11 (7.4%)
A>T	13 (5.1%)	59 (3.4%)	66 (3.6%)	6 (4.1%)
T>A	9 (3.6%)	78 (4.5%)	84 (4.6%)	3 (2.0%)
C>T	64 (25.3%)	416 (23.9%)	431 (23.3%)	49 (33.1%)
G>A	60 (23.7%)	415 (23.8%)	437 (23.7%)	38 (25.7%)
C>G*	6 (2.4%)	80 (4.6%)	85 (4.6%)	1 (0.7%)
G>C	4 (1.6%)	70 (4.0%)	72 (3.9%)	2 (1.4%)
C>A	14 (5.5%)	125 (7.2%)	132 (7.2%)	7 (4.7%)
G>T	24 (9.5%)	136 (7.8%)	151 (8.2%)	9 (6.1%)
MSI-high				
A>C	27 (2.0%)	62 (2.1%)	62 (2.1%)	27 (2.0%)
T>G	23 (1.7%)	59 (2.0%)	59 (2.0%)	23 (1.7%)
A>G	95 (7.1%)	428 (14.4%)	428 (14.4%)	95 (7.1%)
T>C	118 (8.8%)	448 (15.1%)	448 (15.1%)	118 (8.8%)
A>T	32 (2.4%)	58 (2.0%)	58 (2.0%)	32 (2.4%)
T>A	19 (1.4%)	50 (1.7%)	50 (1.7%)	19 (1.4%)
C>T	422 (31.5%)	751 (25.3%)	751 (25.3%)	422 (31.5%)
G>A	432 (32.3%)	811 (27.3%)	811 (27.3%)	432 (32.3%)
C>G	16 (1.2%)	15 (0.5%)	15 (0.5%)	16 (1.2%)
G>C	9 (0.7%)	16 (0.5%)	16 (0.5%)	9 (0.7%)
C>A	74 (5.5%)	157 (5.3%)	157 (5.3%)	74 (5.5%)
G>T	71 (5.3%)	114 (3.8%)	114 (3.8%)	71 (5.3%)

**P* value of 0.010 between intestinal/mixed and diffuse-type carcinomas by Wilcoxon rank sum test (Data from this and a previous study by Wang *et al.*)
